# Scanning electron microscopic features of lacrimal drainage silastic stents: Comparison of various Crawford and large-diameter stents

**DOI:** 10.1371/journal.pone.0295285

**Published:** 2023-12-07

**Authors:** Emmanuel Lee Ong Boniao, Alexander Gerard Nino L. Gungab, Blanche Xiao Hong Lim, Gangadhara Sundar, Mohammad Javed Ali

**Affiliations:** 1 Orbit, Oculofacial Surgery, Department of Ophthalmology, National University Hospital Singapore, Singapore, Singapore; 2 Govindram Seksaria Institute of Dacryology, L.V. Prasad Eye Institute, Hyderabad, India; PGIMER: Post Graduate Institute of Medical Education and Research, INDIA

## Abstract

**Purpose:**

This study aimed to examine the differences in the biofilms and physical deposits on Crawford stents compared to large-diameter stents.

**Methods:**

A prospective interventional study was performed on a series of patients undergoing external or endoscopic dacryocystorhinostomy (DCR) and endoluminal lacrimal duct recanalization (ELDR) with either Crawford or large-diameter stents. All the Crawford stents were retrieved at six weeks and the large-diameter ones at eight weeks following the surgical intervention. There was no evidence of post-operative infection in any of the patients. Following extubation, standard protocols of scanning electron microscopy were used to assess the biofilms and physical deposits on the stents.

**Results:**

A total of 15 stents were studied. Of these, twelve were Crawford, and three were large-diameter stents. The Crawford stents were from two different manufacturers. All the stents demonstrated evidence of biofilm formation and physical deposits. The Crawford stents showed thin biofilms and sparse physical deposits, but there were no demonstrable differences amongst stents from different manufacturers. However, the deposits and biofilms were thicker and more extensive in the large-diameter stents than the Crawford ones. The biofilms from all stents showed the presence of polymicrobial communities within the exopolysaccharide matrix.

**Conclusions:**

The present study found differences in biofilms and physical deposits between Crawford and large-diameter stents. These differences can be partly explained by stent duration, size, and their tissue interactions.

## Introduction

Silicone stents have been commonly used in several lacrimal drainage procedures for decades [1, 2]. There are several types of lacrimal stents, which can be broadly classified as mono and bicanalicular variants. Among the bicanalicular variants, the usage of the Crawford stents is common [3–5]. The large-diameter stents like the STENTube^R^ are believed to reduce the prolapse rate, do not need an endonasal fixation and may help with achieving larger post-operative ostia [6, 7]. They have been used in DCR surgery and in endoluminal lacrimal duct recanalization (ELDR) along with balloon dacryoplasty.

Among the several complications of lacrimal stents, there has been a recent focus on the development of biofilms on the stent surfaces and the possible clinical implications [8–13]. Biofilms are complex communities of microbes living within an exopolysaccharide matrix that helps the organisms reduce their metabolic needs and protects them from anti-microbial agents [9]. Biofilms also help the organism to irreversibly secure themselves to implantable devices like the lacrimal stents. They are known to induce chronic inflammation in vessels (biofilms on venous catheters) and the urinary tract (urinary catheters) [14, 15]. While several studies have looked at the polymicrobial biofilms on lacrimal stents, their actual clinical implications are controversial [8–13]. To further understand the differences based on stent diameters, the present study assessed the physical deposits and biofilms on the surfaces of various bicanalicular stents extubated following the lacrimal drainage procedures.

## Methods

Institutional review board approval of L.V. Prasad Eye Institute was obtained prior to the commencement of this study (LEC 01-14-006). The manuscript adhered to the Tenets of the Declaration of Helsinki. A prospective interventional study was performed on a series of patients (November 2022 to February 2023) undergoing external or endoscopic dacryocystorhinostomy (DCR) with Crawford stents. Written consent was obtained from the patients. Those who underwent endoluminal lacrimal duct recanalization (ELDR) were intubated with Crawford stents, except those patients with diffuse nasolacrimal duct stenosis, where large-diameter stents were used. The Crawford stents (0.64 mm x11 cms stainless steel bodkins) were ‘Lacrimal intubation set or Lacrimal intubation (BVI Visitec^R^, Beaver-Visitec Internation Ltd, Warwickshire, UK or PRICON^R^, Iscon Surgicals Ltd, Jodhpur, India). The large-diameter stents were STENTtube^R^ (0.86 mm central segment and 1.14 mm large diameter segments) (Quest Medical Inc. Allen Texas, USA). All the Crawford stents were retrieved at six weeks and the large-diameter ones at eight weeks following the surgical intervention. There was no evidence of post-operative infection in any of the patients. Following extubation, standard protocols of scanning electron microscopy were used to assess the biofilms and physical deposits on all the segments (ocular and nasal) of the stents [10]. Image Acquisition and analysis were performed using a Zeiss EVO 18^R^(Carl Zeiss Microscopy GmbH, Jena, Germany) at an accelerated voltage of 10 kV and at various magnifications between 82x to 12000x focusing on the external surface of the stent.

## Results

**Fig 1:**
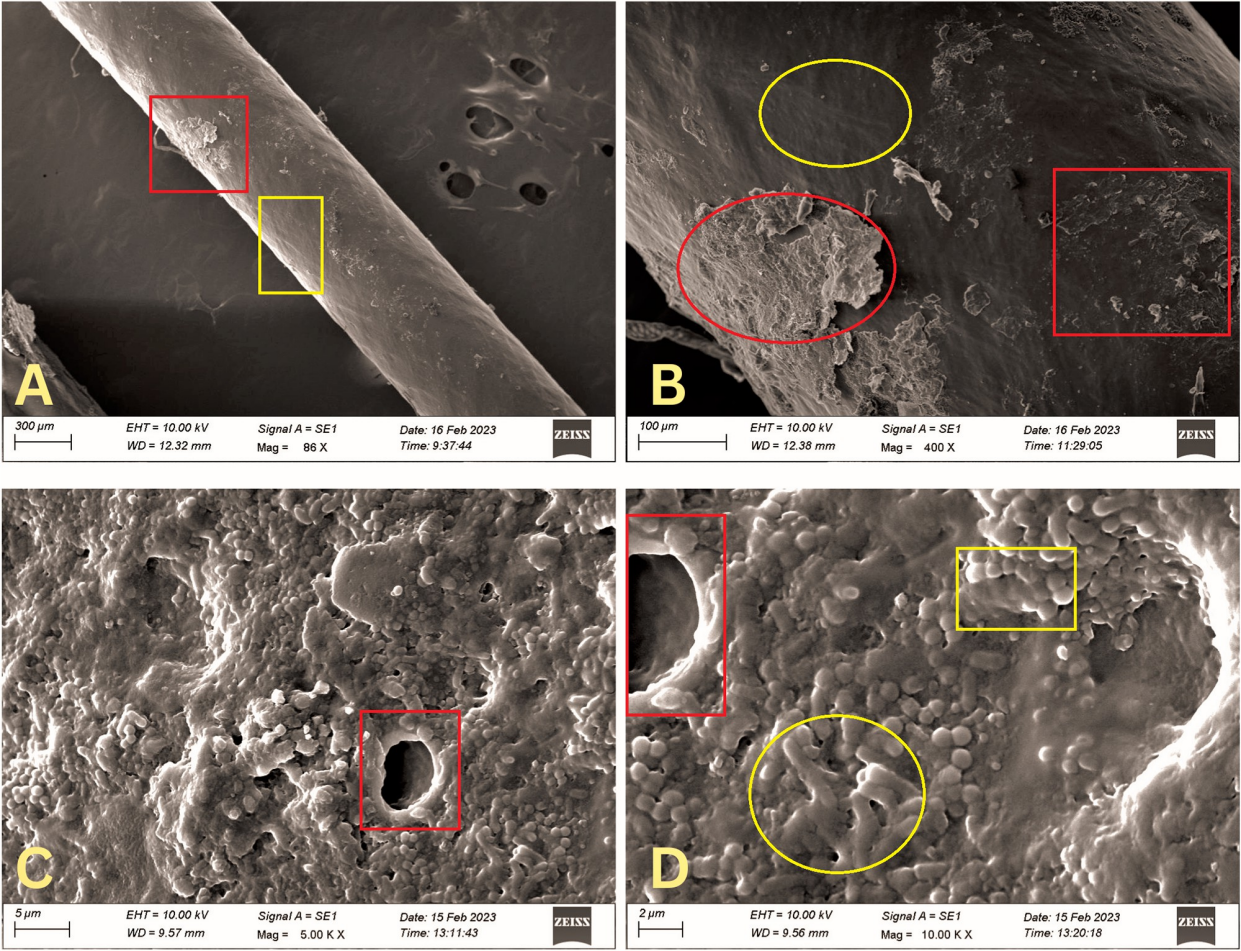
Scanning electron microscopy (SEM) images of the BVI^R^ Crawford stents. Lower magnification image showing patchy physical deposits on the stent surface (red box, **SEM 86x, 1A**). Higher magnification showing focal areas of physical deposits and possibly biofilms. Note the areas of denser deposits (red circle, sparser deposits (red box) and skip areas (yellow circle) (**SEM 400x, 1B**). Very high magnification images demonstrating the characteristic biofilms with exopolysaccharide matrix, water channels (red box) and the polymicrobial communities [coccoid organisms (yellow box) and bacilli (yellow circle] (**SEM 5000x, 1C; SEM 10000x, 1D**).

**Fig 2:**
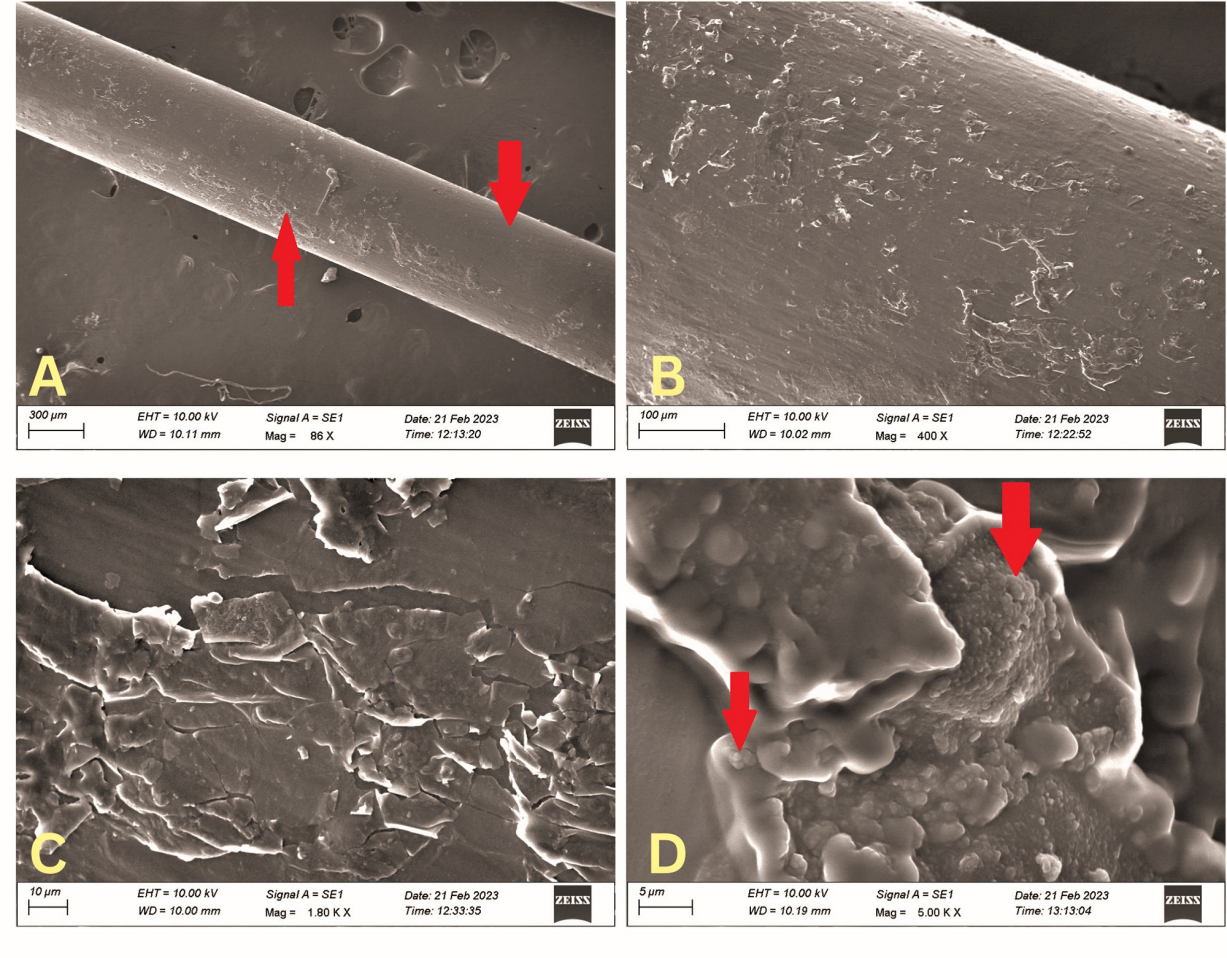
Scanning electron microscopy (SEM) images of the PRICON^R^ Crawford stents. Lower magnification image showing patchy physical deposits on the stent surface (up pointing red arrow) and skip areas (down pointing red arrow) (**SEM 86x, 2A**). Higher magnification showing focal areas of physical deposits and possibly biofilms (**SEM 400x, 2B**). Very high magnification images demonstrating focal areas of physical deposits (**SEM 1800x, 2C**) and the characteristic biofilms with embedded bacterial bodies (red arrows) (**SEM 5000x, 2D**).

**Fig 3:**
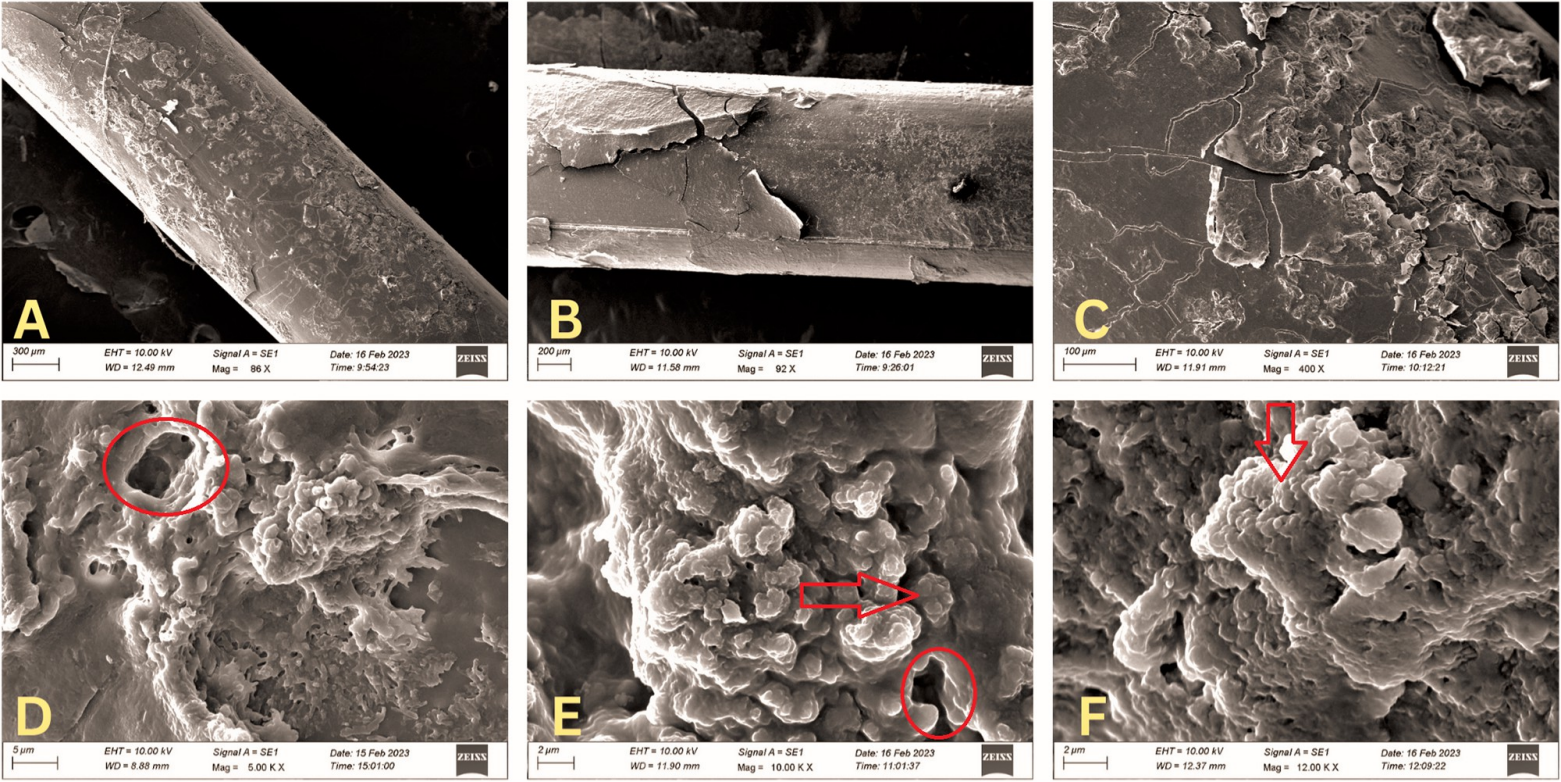
Scanning electron microscopy (SEM) images of the STENTube^R^. Lower magnification image showing dense and diffuse physical deposits on the stent surfaces (**SEM 86x, 3A; SEM 92x, 3B**). Compare these images with those of the Crawford in Figs 1 and 2, at similar magnification. Higher magnification showing focal areas of dense physical deposits and possibly biofilms (**SEM 400x, 3C**) Compare these with similar magnification images 1B and 2B. Very high magnification images demonstrating characteristics of extensive biofilms including the 3D structures, water channels (red circles) and polymicrobial communities (red arrows) (**SEM 5000x, 3D; SEM 10000x, 3E, SEM 12000x, 3F**). Compare it with those in Figs 1 and 2.

A total of 15 stents were studied. Of these, twelve were Crawford (six each of BVI^R^ and PRICON^R^), and three were large-diameter stents. All the stents and all their segments demonstrated evidence of biofilm formation and physical deposits (Figs 1–3). The Crawford stents showed thin biofilms and sparse physical deposits, but there were no demonstrable differences amongst stents from different manufacturers (Figs 1A to 1D and 2A to 2D). However, the deposits and biofilms were thicker and more extensive in the large-diameter stents compared to the Crawford ones (Fig 3A to 3F). The biofilms from all stents showed the presence of polymicrobial communities within the exopolysaccharide matrix (Figs 1D, 2D, 3E and 3F). High-power magnification SEM demonstrated complex 3D exopolysaccharide structures, formation of water channels, and embedded bacterial bodies (Figs 1C, 1D, 2D and 3D–3F) on all the stents.

## Discussion

The present study specifically assessed the development of physical deposits and biofilms on the surfaces of various Crawford stents and compared them to those on the large-diameter STENTube^R^. Although all the stents demonstrated biofilms, the large-diameter stents showed them to be significantly denser and more extensive.

While the Crawford stents are commonly used as intubation devices following lacrimal drainage procedures, the use of large-diameter stents has also increased, though not parallelly. The Crawford stents (BVI Visitec^R^, Beaver-Visitec Internation Ltd, Warwickshire, UK or PRICON^R^, Iscon Surgicals Ltd, Jodhpur, India) have a standard outer diameter of 0.64 mm. The STENTube^R^ (Quest Medical Inc. Allen Texas, USA) is larger in diameter (1.14 mm), nearly twice that of the Crawford. The central segment, which is housed at the medial canthus, is 0.86 mm, still larger than the Crawford. This differential diameter and location facilitate stent security and prevent the complication of stent prolapse [6, 7]. The larger diameter segment, which is housed in the canaliculus and the lacrimal sac (in DCR surgeries) and occasionally in the nasolacrimal duct (ELDR), provides a firm approximation with the tissues and mitigates the need for endonasal fixation or additional knots.

The first report of a biofilm on the stent was in a culture-negative patient who presented with an infection following a dacryocystorhinostomy [16]. Subsequently, there was a demonstration of biofilms on multiple lacrimal stents extubated from post-operative infections with isolation of atypical mycobacteria from 90% of them (n = 10) [8]. While the duration of stent retention could be an important factor, the type of DCR surgery did not appear to influence the development of biofilms [12]. The biofilms have also been implicated in the failure of stent devices in polyurethane lacrimal stents [17]. The biofilm quantification showed the mean biomass on the extubated lacrimal stents to be 0.9385 μm3/μm2 (range: 0.3901–1.9511μm3/μm2) [9]. Different segments of the stents were noted to harbour variable amounts of physical deposits and biofilms [10]. The development of biofilms was well appreciated by four weeks following the intubation [9, 10]. As the duration of the stent retention increases, the biofilms become multi-layered, denser, and more extensive [13]. Since the luminal surfaces of the lacrimal stents were also shown to harbor extensive biofilms, the use of non-luminal stents was proposed [18]. The finding of lacrimal stent biofilms from routine post-operative patients without any signs of infection has led to the debate that the mere presence of biofilms need not necessarily translate to clinical infection. However, its effects on tissue inflammation have not yet been studied.

The present study found that the biofilms on larger diameter stents appeared to be denser, thicker, and more extensive compared to the routine Crawfords stents. This finding need not necessarily reflect the quality of the stent material. Ali et al. in their study on ocular and nasal segments of Crawford stents, found that the ocular segments showed thicker and more extensive biofilms compared to the nasal segments [10]. Interestingly, the thicker biofilms on the ocular surface were focal compared to the diffuse nature of the thinner biofilms on the nasal segment. They attributed this finding to possibly the differential biomechanical movement of the stents in different areas and local stent-tissue interactions. Similarly, compared to Crawford stents, it is possible that the larger diameter tubes move much less within the lacrimal drainage pathway and also have a greater amount of contact with the host tissues. These factors can lead to more physical deposits, and lesser movement of the stent allows less disturbed development of the biofilms.

The limitations of the present study include smaller sample size in each category, lack of biofilm quantification, and discrepancy in the samples of larger diameter stents. The focused indication to use larger diameter stents can explain this discrepancy.

In summary, the present study demonstrated presence of visual differences in the physical deposits and biofilm formation on the surfaces of various Crawford and large-diameter stents. This study can propel further investigations into the biomechanical properties of lacrimal stents and their interactions with the host tissues.
